# Challenges for International Medical Graduates in the US Graduate Medical Education and Health Care System Environment: A Narrative Review

**DOI:** 10.7759/cureus.27351

**Published:** 2022-07-27

**Authors:** Carlos Murillo Zepeda, Francisco Omar Alcalá Aguirre, Edgar Manuel Luna Landa, Edgardo Nahúm Reyes Güereque, Gilberto Pérez Rodríguez García, Lilian Sabinne Diaz Montoya

**Affiliations:** 1 Medicine, Autonomous University of Guadalajara, Zapopan, MEX; 2 Infectious Diseases, National Institute of Medical Sciences and Nutrition Salvador Zubirán, Mexico City, MEX; 3 Surgery, General Hospital of Zone No. 5, Nogales, MEX

**Keywords:** u.s. health care system, u.s. graduate medical education, u.s. medical residency, challenges, foreign medical graduates, international medical graduates

## Abstract

International medical graduates (IMGs) have become a vital part of the US graduate medical education (GME) and health care system (HCS) workforce; they contribute to essential diversity that relieves cultural and linguistic barriers to health care. The number of IMGs looking for medical training in the United States. has constantly been increasing in the last decades. The challenges they meet begin long before residency application, continue during their transition to residency programs, through early medical training, and eventually subside in senior years. IMGs' hurdles permeate the themes of navigating the US GME and HCS, adaptation to the US culture, communication skills, racial discrimination, emotional distress, and finances. This article aims to comprehensively review available information concerning the challenges encountered by IMGs in their transition to the US GME and HCS environments.

## Introduction and background

International medical graduates (IMGs), defined as individuals who received their primary medical degree from a medical school outside the United States and Canada [1], have become a vital portion of the Graduate Medical Education (GME) collective and the US health care system (HCS) and scientific research workforce [2]. They represent a quarter of all training and actively practicing physicians with predominance in primary care specialties [3]. The number of IMGs looking for post-graduate training in the USA has constantly been increasing, as depicted by the 2022 Main Residency Match Results and Data Report in which non-US citizen IMGs experienced a rise of 11% in the total of active applicants since 2018 [4]. In addition, evidence indicates that some IMGs yield better patient outcomes than US practitioners [5]. Also, they outperform US medical graduates (USMG) in the In-Training Examination of internal medicine [6] and score equivalently to USMG residents in general surgery objective assessment [7]. Moreover, IMGs hold more advanced degrees along with added scholarly production than the rest of the applicants [8], contributing to essential diversity that relieves cultural and linguistic barriers to health care in the USA [9].

The challenges confronted by IMGs begin before residency application when they must build a highly competitive profile aiming for a barely equal chance of matching their US peers. Despite higher United States Medical Licensing Examination (USMLE) scores, IMGs have a lower probability than USMGs of being considered for an interview and subsequently being accepted into a residency program; data from previous years computed in the Interactive Charting Outcomes in the Match reveals that the percentage of applicants who matched into internal medicine with a Step 1 score of 220-229 was 99% of USMG seniors and only 51% of non-US citizen IMGs, the latter proportion rises to 89% with a Step 1 score of ≥250 [10]. Thus, factors that increase the probability of securing a residency by IMGs include elevated USMLE scores, US clinical experience through hands-on rotations, letters of recommendation from US physicians, and scientific research [4,11]. In addition, certain program directors exhibit rejection of IMGs during the selection process [12,13]. Upon entry to residency, IMGs meet several events that might preclude a smooth transition to the new work, such as linguistic and cultural barriers, lack of consideration for one's ethnic background, adaptation to settings with different epidemiology and technology tools, and simultaneously grasp new clinical skills and knowledge [11,14-16]. This study aimed to present a narrative description of available information concerning the challenges encountered by IMGs around the transition to the US GME and HCS environment.

## Review

We conducted a PubMed search using the following terms: international medical graduate, foreign medical graduate, needs, challenges, characteristics, discrimination, bias, inclusion, experience, medical residency, Graduate Medical Education, residency program, integration, transition, orientation, acculturation, and wellness; these terms were combined using the Boolean operators: AND, OR. We included experimental and observational studies evaluating both non-US and US IMGs, published in either English or Spanish. To get further information, we scanned relevant articles' references and navigated the website of the Educational Commission for Foreign Medical Graduates (ECFMG), the Accreditation Council for Graduate Medical Education (ACGME), and the American College of Physicians.

Most of the content of this work is constructed using IMGs' subjective experiences and perceptions documented in qualitative studies, complemented with a handful of quantitative studies. We organized this review according to themes originating from the combination of varied resources, including web-based ECFMG modules [17], a presentation offered at the 2020 ACGME Annual Educational Conference [18], a position paper developed by the Canadian Psychiatric Association's Education Committee [19], and a range of studies using grounded theory [15,20-26], critical incident and group focus analysis [27,28], and cogenerative ethnography [29]. We selected the themes present in at least two of the resources mentioned above and incorporated the information into six categories: navigating the US GME and HCS, adaptation to the US culture, communication skills, racial discrimination, emotional distress, and finances. It is essential to know that qualitative-derived themes are not mutually exclusive [20].

IMG challenges

Navigating the US Culture

A study exploring the acculturation of non-US IMGs in a pediatric residency program found that, overall, foreign-trained residents deemed the US life and culture more complex to adjust to than the US HCS, such difficulties comprised housing, daycare, grocery shopping, common sports, and the American school system [28]. Navigating cultural differences is the most commonly reported IMG challenge related to the US GME and HSC transition, registered in up to 17% of residents [30]. Turbulent navigation can occur when engaging in conversation with colleagues or patients about popular US culture topics, including sports and famous people, as this could be troublesome for IMGs, even with prior preparation on the subjects so that dialogues may be restricted to medical issues [22,26]. A major difference noted by IMGs from their home countries was recognizing and managing aspects of family life in the clinical setting; in the US, there is more emphasis on appropriately recognizing and tackling neurodevelopmental disorders of children, child maltreatment, intimate partner violence, and dysfunctional family patterns [23]. Likewise, it might be demanding for pediatric IMG residents to deal with single parents and same-sex couples and collaborate with child protective services, as these encounters could be unusual in their country of origin [28]. IMGs from Asian and Hispanic origins share strong family bonds, and coming to a society with situations that lack a family connection, such as hospitalized geriatric patients receiving no visits and adult children placing parents in remote nursing homes, is alarming [23,29].

Adaptation to the US GME and HCS

Adaptation to the US HCS is second only to cultural barriers as the most common challenge encountered by IMGs in their transition to the USA; its frequency rises to 14% in a recent survey [30]. A study conducted in a pediatric residency program showed that IMGs recognize greater availability of laboratory and imaging tests compared to their home countries. According to the IMGs of this study, the advantages of technology are twofold: ease the diagnostic process and rule out medical conditions that could lead to a lawsuit due to malpractice claims from patients, even when those medical conditions can be detected with elementary clinical skills (defensive medicine) [22,23]. In addition, IMG residents highlight that medical decisions followed by US clinicians rely substantially on patient comprehension and involvement, a model known as patient-centered care, which stands out against the customary unidirectional clinical practice of their country of origin that places physician's opinion above all [25,26]. One reason behind the prevalent patient-centered care in the USA, acknowledged by family medicine IMG residents, is a more profound knowledge of patients' medical conditions [20]. There is also a perception from Japanese IMGs that US patients transfer health care responsibility to clinicians as they would have not only to tell them what to do and arrange follow-up visits but also constantly prompt them to continue care [22].

IMGs coming from backgrounds where long shifts are ordinary and charting is not a significant portion of everyday work may find shocking frequent sign-out schedules and overwhelming the amount of paperwork related to US medical practice [21]; bureaucratic barriers are described by over 9% of IMGs [30]. To illustrate the administrative frustration, Latino IMGs criticized the pressure to accomplish multiple patient visits and that the time spent with them might not be sufficient to provide high-quality care [29]; also, the legal emphasis and formality of documentation are sometimes disturbing [23]. Besides being a novice at charting, many IMGs are unfamiliar with the presentation of clinical cases and the development of personal patient assessment and plans; it is argued that they receive no training on those skills in their medical schools [28]. Within the integrated roles competency, joining teams and understanding each member's function were other distressing tasks for the Middle East and Indian IMGs [22]. Collaboration between health care providers, including physicians, social workers, pharmacists, nurses, physician assistants, physiotherapists, and dieticians, results in expeditious clinical decision-making and patient management, but it could take a great effort from IMGs to move through all the interactions [21,23,28]. Additionally, patient care provided by IMGs can be hampered due to a lack of knowledge about the US health insurance complexity [21].

In the face of the individualistic US learning system that demands active participation to perceive residents' progress and determination [21,31,32], IMG residents encountered difficulties showing decisiveness and expressing one's opinions due to limitations in communication skills, diverse personalities, and the medical education system they are used to, where junior residents do not share their opinion when seniors are present [21,28]. In addition, internal conflict may arise as the fear of being involved in performance feedback because the impression that it represents weakness exposure opposes the accompanying IMGs' aspiration to stand out from the rest to prove themselves to faculty and colleagues [33]. Moreover, there are still countries where academic medical examination modalities include oral, bedside clinical assessments, and written essay exams; this concerns IMGs coming from those places as they must get into the habit of taking standardized multiple-choice board tests to succeed in their residency [29].

A particularly challenging field of the US HCS for IMGs is pain management. It has been noted that foreign physicians' pain control strategies might not be suitable for the standards held in the USA [34]. Also, as they are more likely to meet populations susceptible to suffering from persistent pain, such as the medically underserved, they are likely to see unsatisfactory outcomes in those people [35]. Furthermore, rampant misuse and addiction to opioid analgesics in certain US patients contrast with the prescription practices of controlled drugs followed by IMGs [21], which along with low availability and strict narcotic legislation in their countries of origin, could lead to unsatisfactory pain relief [29].

Communication Skills

Communication is one of the most common challenges identified in studies evaluating the transition of IMGs into the US GME. Around 7% of J-1 visa physician recipients report language or communication barriers [30]. They are more likely to have a native language other than English [36], and, as would be anticipated, the extent of this obstacle depends on participants' prior experience with English communication situations [25]. Dialogue nuances such as accent variations, rapidity of speech, tone, voice inflection, colloquialisms, and local dialect are among described barriers [21,25,28]. On the other hand, patients' understanding of some residents' dialects is also a limitation for effective communication [20].

A critical implication of a lack of English proficiency is that it might increase the risk of misdiagnoses and inadequate patient management, which is especially apparent in the field of psychiatry, where knowledge of the patient's culture and linguistic nuances lead to accurate identification of mental health disorders, this was echoed by Latino residents who become discouraged during such clinical encounters [29]. Further research on behavioral science competency showed that family medicine residents from various countries struggled to identify and attend to every patient's symptoms of potential psychopathologies. Flaws in the medical interview are attributable to little or no prior experience with clinical psychiatry, absence of physician-patient relationship training, and cultural norms or religious beliefs in their home countries [23]. Additionally, IMGs from non-Western countries may have trouble acquiring the attitudes and skills required for competent patient care associated with sexual health due to cultural and religious preconceptions that render sexuality a persistent taboo [28,37]. Another factor related to defective communication in clinical scenarios is that in certain IMGs' cultures, most of the curriculum, if not all, is dedicated to learning hard science without focusing on behavioral disciplines [20]. To expand on that idea, delivering bad news challenged IMGs who are used to releasing that kind of communication to patient's family or friends; many IMGs remark this way of active discussion between the patient and medical staff as a limitation to a fluent decision-making process [25].

It is essential to highlight that Latino residents described how supervisors anticipated a close interaction with shared-language patients, although they came from different countries and cultures [29], and this resulted in the effective closure of a standard gap in the USA, where 18% of the population have a Hispanic origin [38]. There is also evidence that, compared to USMG, IMGs report fewer interpersonal communication difficulties during medical interactions with culturally distinct patients, possibly linked to the effort of the former to overcome cultural differences and their eagerness to connect with patients [32].

Racial Discrimination

Discrimination is a major issue in US GME as it seems that the focus is entirely on the assimilation of the new culture without an appreciation of being different [39]. This phenomenon is seen in all levels of the US HCS, from patients and family members to colleagues and superiors, adding that IMGs have been historically confined to less-desirable specialties and locations [26]. An article published in 1994 revealed the extent of racial discrimination suffered by IMGs in training facilities; up to 23% of participants reported at least one experience of ethnic harassment from patients, attending faculty, peer residents, and nurses. The frequency of self-reported racial incidents was higher in every minority group than in the white population; racial slurs were the most common form of racial discrimination, followed by favoritism and malicious gossip [40]. A more recent report shows cases of IMG exclusion from US colleagues represented by explicit aggressions secondary to contrasting cultures as foreign-born residents were considered unsociable and incompetent [15]. The perception of IMGs' underperformance in clinical scenarios leads to a closer inspection by faculty members and nurse staff compared to their US counterparts [23].

Interviews led by investigators of a fellowship program revealed that not only foreign graduates but also USMG treated as a foreigner (for having accent) are subject to racism [39]. In many cases, discriminatory behavior comes from patients [27], and these events are rarely conveyed to authorities either because offended residents are not aware of policies for reporting patient discrimination or concerned about retaliation [39]. This concealing behavior was recorded in another study assessing residents' transcultural experiences in caring for patients, where discrimination complaints made by IMGs in previous individual interviews were omitted, perhaps voluntarily, following group sessions involving USMG [27]. Some IMGs give an account of the embracing part of the US culture that recognizes and accepts people with diverse origins and opinions [21].

Emotional Distress

Cultural shock leads to mood disorders that interfere with residents' competence [21]. For example, IMGs who have previously completed GME training in their home countries report lower fatigue during the US residency due to duty hour limitations. However, they tend to inform more significant stress and anxiety caused by communication barriers, the feeling of going backward in the medical education hierarchy, loss of family support, the sense of alienation, and frustration for not contributing to changing the shortcomings of their countries of origin that fueled migration [15]. On the other hand, one study found that the self-esteem of Japanese residents seems to improve after the first one to two years, accompanied by an appreciation of personal growth [21]. Similarly, data from a study of community-based internal medicine residency programs evidenced that IMGs score better than USMGs in self-assessed fatigue, personal growth, and self-esteem scales in their transition to residency. The authors of this study suggest that the results obtained are an effect of the energy and enthusiasm of foreign graduates, despite facing significant challenges, in the pursuit of US GME training, and the fact that IMGs hold superior medical school performance than USMG enrolled in less popular specialties such as internal medicine [36].

The presence of other IMGs in the residency program is considered reassuring, especially if they share ethnic backgrounds, as senior residents and program staff can help to overcome administrative, emotional, and academic problems [21]. In addition, IMGs tend to apply to programs located in multicultural areas with large immigrant communities where they become part of a diverse group of residents, and acculturation is common among trainees [29].

Finances

Every step of the journey poses considerable financial stress to applicants. Blackshaw et al. estimated the cost spent by medical students applying to emergency medicine (EM) residency programs, the interquartile range of attending an interview was $185-500, the mean cost of each away rotation was $1,065, and in total, an average of $8,312 would be spent in the pursuit of an EM residency [41]. The article is based on USMGs, so the cost IMGs is estimated to be higher. Although IMGs are less likely than US peers to have high indebtedness at graduation from medical school (OR: 18.3; 95% CI: 5.85-57.26 for a debt of ≤$50K) [36], among the top 10 nations sending applicants for ECFMG J-1 visa sponsorship there are low- and middle-income countries, causing significant economic limitations and making face-to-face proceedings in particular hands-on rotations, obtaining letters of recommendation from US physicians, and becoming familiar with the US GME and HCS conditions affordable only to those with plenty of resources [1,42]. While many residency programs establish US direct patient care experience as a mandatory or preferred requirement [13], not all US teaching hospitals accept IMGs for clinical clerkships; those that do frequently include a long solicitation process, stringent submission requisites, and high application fees [43].

The recent movement to virtual interviews may be more equitable for IMGs; recommendations from the AAMC Interview Guidance for the 2022-2023 Residency Cycle state that virtual interviews from the two previous cycles yielded benefits for all stakeholders. Applicants informed a reduction in travel expenditures and less interference with ongoing responsibilities. In addition, virtual interviewing improves fairness in the selection procedure by removing a big part of the application cost, consequently raising the number of interview invitations accepted from applicants that otherwise could not afford them [44].

After Residency

Finally, residency program admission and adaptation to the new atmosphere are not the only obstacles met by IMGs, most of those who conclude a residency program will seek to remain in the USA and begin independent medical practice. About 1,000 IMGs are recruited annually through the Conrad 30 J-1 Visa Waiver Program to fill vacancies in the Health Professional Shortage Areas (HPSAs) or Medically Underserved Areas (MUA) to avoid the 2-year foreign residence requirement soon after completion of a J-1 visa program [45]. The positions offered through these programs are for primary care specialties, therefore, highly qualified doctors may have to desist from working on the subspecialty they have been training so long for. Around 55-80% of these physicians intend to remain in their communities after the three-year obligation period of the Conrad program [46]. In addition, after the successful accomplishment of medical residency and visa waiver requirements, a substantial number of foreign physicians, up to 81% in a recent survey, consider leaving the HPSA and MUA because of the permanent residency backlog [47]; retention of physicians in shortage areas ranges from 4% to 40% depending on the time spent with original employers [46]. To overcome the expected physician deficit, a deep understanding of the importance and the hurdles of IMGs within the US GME and HCS population is vital [48].

Given the relevance of IMGs and the barriers they tend to face before and after residency admission, many attempts have been made to develop instruments that make them aware of the US HCS and GME environment and support their transition [31,49-62]. The importance and efficacy of qualification programs have been stressed [63], and recommendations for implementing interventions for IMGs have been developed [64]. US clinical experience before applying to residency programs embodies the ideal opportunity to enhance curriculum competitiveness, acquire strong letters of recommendation, and get closer to the national HCS, which can ease the transition to post-graduate training [65]. When away rotations represent a significant economic limitation, and in the event of international restrictions, e.g., the coronavirus disease 2019 (COVID-19) pandemic, standardization of virtual programs that assess IMGs' cultural competence, language proficiency, professionalism as well as clinical skills, and subsequently provide tools for preparing for the US medical residency are needed to promote successful IMG transition and wellness. Successful programs are those that not only address individual needs of IMGs but also provide counseling to peers and supervisors during and after implementation [64].

## Conclusions

IMGs find the US medical residency an opening to go beyond the limitations of their home countries and access high-quality GME. However, they must deal with all the associated challenges as part of the journey. Different countries of origin and educational backgrounds lead to varied perceptions and challenges around the transition to US GME; however, clusters of everyday experiences allow for the development of resources directed at well-defined categories. The transition of IMGs to the US GME and HCS is full of challenges that can be effectively confronted with orientation courses before and after entry to residency, wide availability of those programs would represent a significant aid to incoming IMGs. Studies included in this review have restricted generalizability as they are predominantly qualitative, and sampling is limited to small groups. The development of large-scale quantitative studies evaluating the prevalence of the challenges mentioned above within residency programs would underline the actual extent of the problem.
